# Paediatric Behçet’s Disease: A Comprehensive Review with an Emphasis on Monogenic Mimics

**DOI:** 10.3390/jcm11051278

**Published:** 2022-02-26

**Authors:** Ovgu Kul Cinar, Micol Romano, Ferhat Guzel, Paul A. Brogan, Erkan Demirkaya

**Affiliations:** 1 Department of Paediatric Rheumatology, Great Ormond Street Hospital for Children NHS Foundation Trust, Great Ormond Street, London WC1N 3JH, UK; ovgu.kulcinar@gosh.nhs.uk (O.K.C.); paul.brogan@gosh.nhs.uk (P.A.B.); 2 Division of Medicine, National Amyloidosis Centre, Centre for Amyloidosis and Acute Phase Proteins, University College London, Royal Free Campus, Rowland Hill Street, London NW3 2PF, UK; 3 Department of Pediatrics, Division of Pediatric Rheumatology, Schulich School of Medicine & Dentistry, University of Western Ontario, London, ON N6A 5W9, Canada; micol.romano@lhsc.on.ca; 4 Canadian Behcet and Autoinflammatory Disease Center (CAN-BE-AID), University of Western Ontario, London, ON N6A 4V2, Canada; 5 Molecular Genetics Laboratories, Department of Research and Development, Ant Biotechnology, Istanbul 34775, Turkey; frhtgzl@gmail.com; 6 Great Ormond Street Institute of Child Health, University College London, 30 Guildford Street, London WC1N 1EH, UK; 7 Department of Epidemiology and Biostatistics, Schulich School of Medicine & Dentistry, University of Western Ontario, London, ON N6A 5W9, Canada

**Keywords:** A20 haploinsufficiency, Behçet’s disease, inflammatory vasculitis, monogenic mimics, next-generation sequencing, NF-κB pathway, paediatric Behçet’s, whole-exome sequencing

## Abstract

Behçet’s disease (BD) is a polygenic condition with a complex immunopathogenetic background and challenging diagnostic and therapeutic concepts. Advances in genomic medicine have provided intriguing insights into disease pathogenesis over the last decade, especially into monogenic mimics of BD. Although a rare condition, paediatric BD should be considered an important differential diagnosis, especially in cases with similar phenotypes. Emerging reports of monogenic mimics have indicated the importance of genetic testing, particularly for those with early-onset, atypical features and familial aggregation. Treatment options ought to be evaluated in a multidisciplinary setting, given the complexity and diverse organ involvement. Owing to the rarity of the condition, there is a paucity of paediatric trials; thus, international collaboration is warranted to provide consensus recommendations for the management of children and young people. Herein, we summarise the current knowledge of the clinical presentation, immunopathogenetic associations and disease mechanisms in patients with paediatric BD and BD-related phenotypes, with particular emphasis on recently identified monogenic mimics.

## 1. Introduction

Behçet’s disease (BD), initially described by Hulusi Behçet, is a multisystem inflammatory vasculitis of unknown aetiology with the clinical triad of aphthous stomatitis, genital ulceration and uveitis [1]. In the following years, vascular involvement with thrombophlebitis and multisystem features with vital organ involvement were also identified [2,3]. The disease is now characterised by recurrent oral and genital ulceration, inflammatory eye disease and vascular, cutaneous and neurological involvement. In the International Chapel Hill Consensus Conference (CHCC) 2012, Behçet vasculitis was reclassified as a ‘variable’ type, given that it may affect all types and sizes of vessels, although veins are predominantly involved [4]. Disease onset is usually between the second and fourth decades of life and is twice as common amongst males, who generally have a more severe phenotype. More recently, with the increased awareness of BD in different populations, paediatric presentation before age 16 has been reported to account for 3.6–26% of cases [5,6].

BD is characterised by exacerbations and remissions; additionally, disease onset is generally insidious, with the slow accrual of effects on different systems over months or years, and thus, delayed diagnosis continues to be a major concern [7]. Given the rarity of the disease, the lack of laboratory diagnostic tests and the high frequency of non-specific features such as headaches, arthralgia and abdominal pain in children, BD diagnosis fundamentally depends on clinical judgement [8,9]. Although BD is considered a single disease entity, clinical features at presentation may broadly vary depending on gender, geographical area and age of onset [10,11]. The underlying mechanisms of BD are yet to be fully delineated. Recent discoveries of monogenic mimics of BD such as A20 haploinsufficiency emphasise the prominence of Mendelian genetic factors, especially for younger patients, but on the whole do not provide clear insights into the pathogenesis of typical (i.e., non-monogenic) BD [12,13].

In light of the expanding knowledge on BD immunopathogenesis and recent advances in genomics, herein, we provide a critical analysis of the literature with an emphasis on aetiopathogenesis, genetic background, BD mimics and clinical evidence for the current pharmacological treatments available for paediatric BD.

## 2. Epidemiology

### 2.1. Incidence and Prevalence

The prevalence of BD in adult patients varies geographically, with increased frequency in populations living along the Silk Road from Japan to the Eastern Mediterranean basin, considered to be endemic areas for the condition [14]. Although BD is more common in Turkey and other parts of the Middle East, increased awareness of the condition in other countries has resulted in increased recognition outside of these endemic areas [14]. In a meta-analysis, pooled estimates of prevalence in adult BD patients were 119.8 per 100.000 in Turkey (95% CI: 59.8, 239.9), 31.8 per 100.000 in the Middle East (95% CI: 12.9, 78.4) and 3.3 in 100.000 in Europe (95% CI 2.1, 5.2) [15]. Ozen et al. demonstrated that the prevalence of BD in Turkish children is not more than 10 cases per 100.000 [16], whereas more recently, a prospective epidemiological study conducted in children and young people with BD younger than 16 years of age in the UK and Ireland showed that the estimated two-year prevalence was 4.2 per million children (95% CI: 3.2, 5.4) [9]. BD is reported to occur more rarely in Western European countries, with a prevalence of 0.1 in 100.000 in Sweden, 7.1 per 100.000 in France and 15.9 per 100.000 in Southern Italy [17,18,19]. In addition to country of residence, ethnicity was shown to be an important factor in disease prevalence. In a French epidemiological study, BD rates were significantly higher in immigrants of North African and Asian ancestries living in the Paris area as compared to those of European origin [11]. Interestingly, disease rates in immigrant populations were not as high as those among people who lived in their home countries, indicating the role of environmental factors in disease pathogenesis. Likewise, geographical variation in disease expression is also suggestive of environmental effects and epigenetics [14]. For example, gastrointestinal manifestations are more common in Japan but rare in Turkey. In contrast, vascular involvement is more frequent in Turkey and Middle Eastern countries as compared to the Far East [14].

### 2.2. Demographics and Clinical Features

The mean age of onset in paediatric BD significantly varies between different studies, from 4.87 to 12.3 years [6,20]. Children and young people with BD generally initially present with fewer symptoms than required to fulfil diagnostic criteria, and therefore, the time from the first symptom to diagnosis is approximately 3–5 years [6,10,21]. Recent epidemiological studies tend toward an equal female-to-male sex ratio. Though study results have been conflicting, it is important to note that significant symptom variability has been shown between genders in different studies, with increased disease severity in males, including more frequent vascular and ocular involvement [10,21,22]. Of note, genital ulceration was reported to be more common in females in some studies [9,20,23].

## 3. Aetiopathogenesis

### 3.1. Aetiology and Immunopathogenesis

The aetiopathogenesis of BD is still unclear; however, both innate and adaptive immune responses, along with environmental factors and genetic predisposition, are considered important [24]. Environmental triggers may include bacterial infections, viral infections or responses to autoantigens. Streptococcus is commonly isolated from BD patients, and Streptococcus sanguis, Helicobacter pylori, Mycoplasma and herpes simplex virus 1 are some of the microbial agents investigated in the BD aetiology [24]. Oral infections, which were proposed to be associated with the disease by Behçet himself, are still considered to be potential triggers [1]. In addition to microbial agents, autoantigens such as heat-shock proteins (HSP) have been shown to play a role in the development of the disease by acting as molecular mimics [24].

There have been attempts to classify BD under autoimmune diseases, seronegative spondyloarthropathies and, more recently, autoinflammatory diseases (AIDs) [25]. Evidence supporting the classification of BD in the autoimmune disease group stems from the following observations: major histocompatibility complex class I (MHC-I) identification, response to immunosuppressive treatments and the presence of antigen-specific T lymphocytes, such as T-helper 17 (T_h_17) pathway involvement and IL-17 and IL-21 production from T_h_17 cells [24,26]. Ombrello et al. [27] provided additional insights into the debate regarding BD classification, with their data showing that MHC class I molecules could increase the risk of developing BD through the regulation of both innate NK cells and adaptive cytotoxic T lymphocytes. On the other hand, there are also some limitations of the classification of BD under the autoimmunity category. Firstly, the absence of classical autoimmune features such as female predominance, the lack of association with other autoimmune diseases and the absence of B cell involvement and autoantibody production are notable [28]. Secondly, the presence of innate immune cells, namely, neutrophils and NK cells, and proinflammatory cytokines and chemokines secreted upon activation of these cells may explain unprovoked hyperinflammatory episodes, which would indeed fit well with an autoinflammatory condition of multifactorial origin [14,28]. Additionally, a small number of patients with BD were found to be carriers of mutations in MEFV (encoding the pyrin protein that plays a key role in the inflammasome complex and whose mutations cause familial Mediterranean fever), and some had variants in genes encoding Toll-like receptors (TLR-4) of the innate immune system, further directing BD toward an autoinflammatory pathogenesis [29]. Nevertheless, several important factors need to be taken into consideration before drawing precise conclusions: (a) most AIDs present early in life and are associated with a monogenic mutation, which is not the case in BD [30]; (b) vasculitis is not a prominent feature of monogenic AIDs (with some notable exceptions), whereas a substantial number of BD patients display vascular involvement at presentation or over the disease course [30]; (c) anti-IL1ß therapies that have been successfully used in AIDs caused by defects in the inflammasome have not been convincingly effective for BD [14]. Taken together, given the complexity in aetiopathogenesis and current evidence supporting the involvement of both innate and adaptive immunity, it is difficult to classify BD as either exclusively autoimmune or autoinflammatory.

### 3.2. Genetic Background

BD is a multifactorial polygenic disease. Disease epidemiology and possible explanations for underlying pathogenic mechanisms suggest a substantial genetic influence [31]. Indeed, the peculiar geographical distribution, cluster of symptoms and familial aggregation of the disease in both paediatric and adult populations (including increased sibling and twin recurrence rates), with frequencies ranging from 10 to 50%, strongly indicate an association with different genetic factors [31,32].

**Figure 1:**
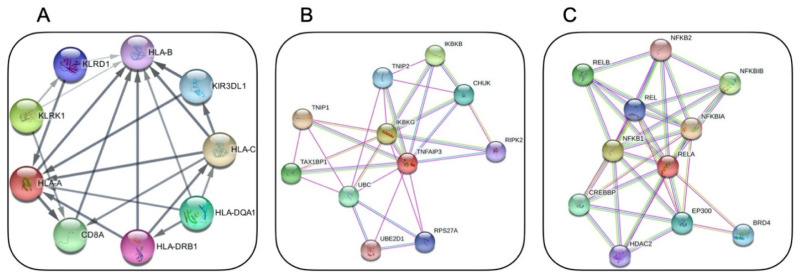
STRING app [39] physical subnetwork visualisation with Cytoscape Software [40] for: (**A**) HLA-B from within Behcet disease–related genes (the resulting network consists of 21 functional associations between 9 proteins with a confidence cut-off of 0.4 (PPI enrichment *p*-value: 7.02 × 10^−^^7^)); (**B**) A20 protein (the resulting network consists of 29 functional associations between 11 proteins with a confidence cut-off of 0.4 (PPI enrichment *p*-value: 0.000522)); (**C**) RELA protein (the resulting network consists of 36 functional associations between 11 proteins with a confidence cut-off of 0.4 (PPI enrichment *p*-value: 1.11 × 10^−^^7^)).

Recent advances in genome-wide association studies (GWASs) and next-generation sequencing as diagnostic tools have provided more insights into innate and adaptive immune regulation, which in turn shed light on the genetic background of BD [33]. Human leukocyte antigen (HLA) loci, most importantly, HLA-B*51, have long been demonstrated to be the strongest genetic susceptibility factor for BD, with the replication of results in different populations [34] (Figure 1A). Although HLA-B*51 remains the most significant association, GWASs were able to identify further susceptibility loci in BD. In a GWAS by Remmers et al. [35], 1215 BD cases and 1278 healthy controls were evaluated, and the results not only supported the association of HLA-B*51 as the primary susceptibility factor for BD but also revealed another independent MHC class I association. The same study also demonstrated that individuals with variations in the IL-10 and IL23R/IL12RB2 gene regions were predisposed to BD [35]. Of note, variations in the IL23R gene region, encoding an upstream molecule in T_h_17 activation, have been associated with ankylosing spondylitis, psoriasis and inflammatory bowel disease, all of which share some phenotypic overlap with BD, supporting the viewpoint that it should be classified under seronegative spondyloarthropathies [36]. Following this, Kirino et al. [37] reported another GWAS evaluating 779,465 single-nucleotide polymorphisms (SNPs) in 1209 Turkish BD patients and 1278 healthy controls, resulting in the identification of novel genetic variants in STAT4 (signal transducer and activator of transcription 4) and ERAP1 (endoplasmic reticulum amino peptidase 1). ERAP1 functions to cut peptides in the endoplasmic reticulum before they are loaded onto MHC class I for antigen presentation, suggesting that peptide processing and binding/presentation mechanisms of adaptive immunity contribute to the disease pathogenesis [37]. Furthermore, the interaction between HLA-B*51 positivity and ERAP1 homozygosity was associated with an odds ratio (OR) for BD of 3.78 (95% CI: 1.94–7.35) versus an OR of 1.48 (95% CI: 0.78–2.80) in HLA-B*51-negative individuals. Having said that, HLA-B*51 is not useful as a diagnostic test for BD due to its rarity in other populations as compared to endemic regions and due to its high prevalence in healthy individuals [8,38].

Relatively recently, a novel AID with a familial BD-like phenotype was identified in association with germline loss-of-function mutations in the *TNFAIP3* gene, leading to haploinsufficiency of the A20 protein, which is a regulatory protein in the NF-κB signalling pathway [13] (Figure 1B). Consequently, mutant A20 proteins could not restrict NF-κB activation, resulting in the overstimulation of inflammatory pathways and overproduction of inflammatory cytokines, such as IL-1β, IL-6 and TNF-alpha, thereby causing an autoinflammatory phenotype in these patients. In a recent study involving 16 patients with A20 haploinsufficiency, disease onset was mostly early in life (range: first week of life to 29 years of age); in addition, oral, genital and/or gastrointestinal ulceration were hallmark features, mimicking BD [41].

Further reports of monogenic mimics of BD have continued to emerge over the last decade. Papadopoulou et al. [33] reported 11 paediatric cases that were referred as suspected BD and ultimately diagnosed with an underlying monogenic disease (the median age at the disease onset: 0.6 years (range: 0.2–2.3 years); the median age at the first clinical visit: 4.1 years (IQR: 2.2–4.8 years)). A wide variety of final diagnoses involving A20 haploinsufficiency, *MEFV* mutations, chronic granulomatous disease and other monogenic immunodeficiencies were reached in their cohort, leading the authors to conclude that monogenic mimics should always be considered in the workup of paediatric BD.

Collectively, these studies suggest that early presentation (<5 years of age), strong family history and/or incomplete or atypical clinical features (even in older patients) of BD should be considered ‘red flags’, and monogenic AID or primary immunodeficiency should be screened for early in the workup [13,33].

## 4. Diagnostic Criteria

The definition of diagnostic criteria for BD has been a challenging task, given the complexity of the condition, geographical differences in symptom expression and the clustering of symptoms. The most frequently used diagnostic criteria for BD are the ‘International Studies Group (ISG)’ and revised ‘International Criteria for BD (ICBD)’ criteria [42,43] (Table 1). Since the performance of these criteria was suboptimal in paediatric practice, more recently, an international group of paediatricians proposed paediatric BD classification criteria (PED-BD) from their large cohort of paediatric BD patients [23] (Table 1). In PED-BD, recurrent oral ulceration is not mandatory, although it is one of the criteria, along with genital ulceration, skin, eye, neurological and vascular involvement. The pathergy test has been excluded from PED-BD due to its low specificity. The presence of three out of six criteria is required for a diagnosis of paediatric BD [23]. In general, the literature often confusingly refers to these criteria as either diagnostic and/or classification, although diagnostic and classification criteria are not one and the same. Classification criteria are defined as a set of disease features used to categorise patients into specific, nearly homogeneous groups with similar disease features [44,45]. They are designed to guide clinical trials and further research of homogeneous patient cohorts to understand disease pathogenesis and to assess treatment responses; thus, they should not be used for diagnostic purposes in individual cases [44,45]. On the other hand, diagnostic criteria are a combination of clinical signs, symptoms and laboratory, imaging or histopathology tests that can be used to diagnose an individual patient [44]. In BD, several diagnostic and classification criteria have been proposed, yet there is a paucity of established consensus criteria in the paediatric population. Acknowledging this gap, PED-BD classification criteria were developed to identify subgroups of paediatric cases to better understand the clinical course of the disease and to guide future clinical trials [23]. Notably, the performance of the PED-BD criteria is still being evaluated in different cohorts, and current evidence suggests that ICBD is the most sensitive criteria set [38]. A comparison of the most frequently used ISG and ICBD diagnostic criteria, along with the consensus PED-BD classification criteria, is provided in Table 1.

## 5. Clinical Manifestations

The heterogeneity of presenting symptoms is one of the most striking features of BD and characterised by unpredictable exacerbation and remission episodes [14]. Considerable variability in clinical expression might be explained by age of onset, geographic location and ethnicity. Patients from the Far East exhibit more gastrointestinal symptoms as compared to those of Mediterranean origin [46]. Another interesting aspect of BD is the distinct clustering of symptoms, such as the acne–arthritis–enthesitis phenotype or the vascular subset, which indicates the complexity of the disease and further suggests the involvement of more than one immunopathogenetic mechanism [25]. The most common clinical features in both paediatric and adult cases—and usually in presenting symptoms—are mucocutaneous involvement with oral and genital ulcers and skin manifestations [6,10,20,47,48]. Ocular, neurological and vascular manifestations were reported to be less common among paediatric cases but more severe when they do occur [49]. Current evidence suggests a time interval between the first clinical manifestation and the development of other BD features, which may result in delayed diagnosis, particularly in younger patients [9,50]. In a cohort of 817 BD patients from France [51], vasculitis with mainly arterial involvement, male sex and a high number of disease flares were associated with increased risk of mortality.

### 5.1. Mucocutaneous Involvement

The majority of paediatric patients present with painful recurrent oral ulcers (ROUs), which may precede disease onset and can appear on the tongue, lips, palate and cheeks [9,10,52]. They are usually round, discrete lesions with a yellow-grey pseudomembranous base and heal without scarring [8]. The differential diagnosis for isolated ROU involves a wide range of diseases, potentially including autoimmune diseases, inflammatory bowel disease (IBD), immunodeficiencies and autoinflammatory syndromes [8].

Genital ulcers (GUs) are less common in children than in the adult population, occurring in 55–83% of patients [9,23]. GUs can be extremely painful, and, in contrast to ROUs, they tend to be deeper and leave a scar. Typical localisations are the scrotum, glans penis, vulva, vagina and perianal region; hence, the condition needs to be distinguished from IBD [8].

BD can cause a wide range of skin manifestations that were shown to be very frequent among paediatric cases, reported in approximately 90% [10]. Cutaneous manifestations may involve acneiform (papulopustular) lesions, necrotic folliculitis (more common in males), erythema nodosum (more common in females), purpura and ulcers.

The pathergy phenomenon, which can be defined as a non-specific cutaneous pustular reaction and is performed by needle puncture of the dermis, was formerly used to show skin hypersensitivity within 24–48 h of the injury. Due to significant variability in the positive test results between study groups and populations (from 14.5% to 80%), it is now neither regarded as a diagnostic test in adult patients nor used as a criterion for paediatric BD [3,23,42,47].

### 5.2. Musculoskeletal Involvement

Musculoskeletal (MSK) involvement can present as oligo- or polyarticular non-erosive inflammatory arthritis or arthralgia in children [20,23,48]. Although knees and ankles are reported to be the most frequently affected joints, spondyloarthropathy and enthesopathy can also be observed, which is consistent with reports suggesting the classification of BD under seronegative spondyloarthropathies or MHC-I-opathies [14].

Although myositis is rarely associated with BD, both generalised and localised myositis have been reported in paediatric BD [52,53].

### 5.3. Ocular Involvement

The most common ocular manifestations of BD include anterior and posterior uveitis, panuveitis, retinal vasculitis and retinitis [8,23,47,54], the prevalence of which varies significantly, from 8.7% in the United Kingdom to 66.2% in an Iranian paediatric cohort [20,48]. Anterior uveitis is reported to be more frequent in patients younger than 10 years old, while panuveitis tends to occur later in the disease course [23]. Complications may involve cataracts, maculopathy, posterior synechiae, retinal detachment and optic atrophy [54]. In a recent prospective paediatric BD cohort, visual loss due to severe uveitis and enucleation was reported [9]. Male sex was found to be associated with an increased rate and severity of ophthalmic involvement in BD [10,54].

### 5.4. Vascular Involvement

BD can affect vessels of any size and type; hence, it is classified as ‘variable’ vasculitis in the revised Chapel Hill consensus on the nomenclature of vasculitides [4]. Inflammation of the vessel wall resulting in thrombus formation is usually the main pathologic feature. In the consensus classification of paediatric BD, vascular involvement (one of the criteria) refers to venous and arterial thromboses and/or arterial aneurysms [23]. Vascular manifestations were reported in 6.5% to 32% of patients in paediatric cohorts from various countries [20,23,55,56]. Similar to ocular symptoms, vascular signs were also found to be more common in males in different studies [23,55,56]. In a retrospective chart review of 21 paediatric cases with at least one episode of thrombosis, cerebral venous sinuses were most frequently affected (in more than half of patients), followed by lower extremities [57]. In the same study, thrombophilia measurements were unremarkable in the majority of the cases (14/21), while 4/21 had positive anticardiolipin antibodies, 2/21 had protein C deficiency and 1/21 had a positive lupus anticoagulant test [57]. In a retrospective study in Turkey, 11/34 (32%) paediatric BD patients developed vascular involvement during follow-up [55]. Amongst these 11 patients, 9/11 had only venous involvement, 1/11 had only arterial involvement and 1/11 had both arterial and venous involvement [55]. In addition, 6/11 were reported to have neurovascular involvement, while 2/11 developed pulmonary artery aneurysms [55]. Cohorts from the UK [9,20] and Italy [56] showed less frequent vascular signs in paediatric BD—mainly with venous thrombosis of the central nervous system or lower extremities [14]. In an international collaborative study with 86 paediatric BD cases from Turkey, France, Saudi Arabia and Iran, 10 episodes of venous thrombosis in 8 patients were observed; lower extremities, inferior vena cava and cerebral vessels were the most commonly affected areas [10]. Arterial complications with thrombosis and aneurysms occurred in six patients, and pulmonary arteries were the most frequently affected, resulting in haemoptysis [10]. In another study involving 12 paediatric cases with central venous sinus thrombosis (CVST), headache was universal at presentation (100%), followed by vomiting (25%) and blurred vision (16.7%) [58]. The transverse venous sinus was affected in the majority (75%), followed by the superior sagittal sinus [58]. Of 12 patients, 4 had another venous thrombosis in addition to CVST [58]. Likewise, in a cohort of 26 paediatric neuro-BD cases, CVST was confirmed in 23/26, the majority of whom presented with headaches [59].

### 5.5. Central Nervous System Involvement

Central nervous system (CNS) involvement is reported in 5% to 30.9% of cases in paediatric cohorts [10,20,56,60]. Both parenchymal and non-parenchymal vascular lesions can lead to CNS manifestations in paediatric and adult BD [59]. In a large cohort with 26 paediatric neuro-BD cases, 3/26 had parenchymal involvement, which was confirmed by clinical examination and magnetic resonance imaging (MRI) [59]. Although non-specific headaches are the most common presenting symptom in paediatric neuro-BD, meningoencephalitis, idiopathic benign hypertension and cranial nerve palsies have also been reported; therefore, detailed neurological examination is warranted [10,56]. In the aforementioned international cohort study by Kone-Paut et al. [10], other CNS features were meningitis (9%), benign intracranial hypertension (4%), hemiparesis or paraparesis with spastic quadriparesis (4%), seizures (3%) and peripheral neuropathy including sixth nerve palsy. Severe disability was reported in 8/10 patients with paediatric neuro-BD from the same cohort, attributed to meningoencephalopathy or other neurologic deficits [10]. Four of the heavily affected neuro-BD children also developed organic psychiatric disorders, including memory loss and personality changes for one patient. In a UK cohort of 28 paediatric BD patients [20], 13 neuro-BD cases were identified, of which 11/13 reported frequent (more than once a week) headaches, 1/13 had seizures and 1/13 developed neurogenic bladder dysfunction during the disease course [20]. Computed tomography and/or MRI of the brain was performed in 6 cases; in 2/6 patients, sagittal sinus thrombosis was detected, while 4/6 had normal imaging [20]. In a recent prospective study, sensorineural deafness was also reported in one patient with paediatric BD [9].

### 5.6. Gastrointestinal Involvement

Gastrointestinal manifestations wax and wane in paediatric BD. Abdominal pain, gastritis and diarrhoea have been reported as the most frequent gastrointestinal symptoms in different studies [10,20,23,56]. Ulcerative lesions of the gastrointestinal tract and perianal region, haematemesis, melaena, hepatomegaly and gastric perforation were also reported, albeit infrequently [23,55,56]. In a study by Nanthapisal et al. [20], gastrointestinal tract endoscopy performed in 10 children revealed gastritis (*n* = 3), colonic ulcers (*n* = 2), oesophagitis (*n* = 1) and oesophageal ulceration (*n* = 1), while the remaining 3 had no endoscopy findings. Hepatic vein occlusion may lead to Budd–Chiari syndrome, as reported in one patient in the international cohort studied by Kone-Paut et al. [10].

### 5.7. Other Uncommon Manifestations

Constitutional symptoms with recurrent unexplained fevers, malaise, fatigue and mood disorders have been reported [56].

#### 5.7.1. Nephrourological Involvement

Urinary sediment changes with mild proteinuria, haematuria, glomerulonephritis and tubulointerstitial nephritis have been observed in a small number of paediatric BD cases [9,10,20,56]. Secondary amyloidosis is extremely rare in children, though documentation exists in adult cohorts [61]. Considering the paucity of data in paediatric cases, further follow-up studies with international collaboration are needed to identify patients at risk of developing secondary amyloidosis. Urological manifestations with epididymo-orchitis and urethritis were also reported in some larger cohorts [10,48].

#### 5.7.2. Pulmonary and Cardiac Involvement

Pulmonary arterial aneurysms may rarely occur in some paediatric cases and can present with life-threatening haemoptysis, dyspnoea, cough and chest pain [10,55]. Although very rare in paediatric BD, cardiac involvement with endocarditis, myocarditis, pericarditis, pericardial effusion and arrhythmias has been reported [10,20].

## 6. Management and Outcome Measures

Management of BD requires a multidisciplinary team approach due to the heterogeneity of symptoms with diverse organ involvement. The main goal is to suppress inflammatory exacerbations, prevent organ damage and maintain optimal quality of life [14]. Achieving this goal can be challenging because of the lack of standardised outcome measures defining remission [14]. A variety of outcome measures have been used in clinical trials in the adult population, albeit without validation [62].

Considerable variation in presenting features depending on age, sex, symptom onset and geography has also led to the development of diverse outcome measures, particularly in adult BD patients [62]. This has made it difficult to compare study results and optimise management strategies. In a systematic review evaluating outcome measures used in adult BD trials, the most commonly used tools for assessing disease activity or severity were: (1) the Behcet’s Disease Current Activity Form (BDCAF); (2) the Iranian BD Dynamic Activity Measure (IBDDAM); and (3) Krause’s total severity score for disease severity [62]. In a study assessing the performance of outcome measures in paediatric BD, the Physician Global Assessment (PGA) score showed a positive correlation with BDCAF and IBDDAM, indicating the potential use of these measures for future paediatric trials [63]. The lack of unified and reliable outcome measures for BD in both paediatric and adult populations has been a major obstacle in designing randomised controlled trials.

EULAR consensus recommendations for adult BD were published in 2018 [64], but due to the rarity of BD in children, heterogeneity of disease expression and lack of randomised controlled studies in the paediatric population, there are currently no consensus management guidelines for paediatric BD. Management and treatment strategies have therefore been based on adult trials and adult cohorts. Topical corticosteroids followed by colchicine are historically reported as the first-line therapeutic options to control orogenital ulcerations in the majority of adult and paediatric cases [9,20,21,55,56]. Treatment escalation and the choice of medication fundamentally depend on organ and system involvement, but in general, the use of disease-modifying anti-rheumatic drugs (DMARDs), namely, azathioprine, mycophenolate mofetil, methotrexate or sulphasalazine, have been reported in different countries [9,55,56]. In refractory cases or cases of severe organ involvement, anti-TNF and other biologic agents such as anti-interleukin 1 (anakinra or canakinumab), anti-interleukin 6 receptor (tocilizumab) or interferon-alpha have also been used with varied success [9,20,55,56]. Of note, no controlled trials have been conducted to show the efficacy of colchicine or other medications in paediatric BD.

### 6.1. Management of Orogenital Ulcerations and Skin and Joint Involvement

Topical treatment with corticosteroids and sucralfate can be used for mucocutaneous lesions, especially if recurrences are infrequent and if they do not impair quality of life. In patients with frequent recurrences, systemic treatment with short courses of corticosteroids can be considered, although, in this setting, colchicine would be the first choice of treatment, considering its safe side-effect profile, low cost and the possibility of avoiding steroid toxicity [8,12,14,20]. Colchicine has been shown to be more effective in controlling genital ulcers, arthritis and erythema nodosum as compared to oral lesions [65], but it has been widely used and suggested as a first-line systemic option in mucocutaneous disease in EULAR consensus recommendations [64].

The successful use of thalidomide was reported in five children with the improvement of orogenital ulcers; however, peripheral neuropathy was documented in two patients [66]. The high risk of peripheral neuropathy and teratogenicity has limited thalidomide use [66], which has largely fallen out of favour with increased use of anti-TNF. Dapsone has been another treatment option to control mucocutaneous symptoms in adult BD cases [67], although haemolytic anaemia is an important adverse side effect of this treatment. Additionally, there is a lack of studies regarding the use of dapsone in paediatric BD.

An oral phosphodiesterase-4 inhibitor, apremilast, has been successfully used in treating oral and genital ulcerations in the adult population [68].

In colchicine-resistant patients, systemic immunosuppressive therapeutic options including azathioprine, anti-TNF or apremilast can be considered [8,12]. A randomised controlled, double-blind trial of azathioprine in the adult population demonstrated that it was effective in controlling orogenital ulcerations, arthritis and eye disease without causing serious adverse events [69]. It is important to emphasise that there have been no controlled trials of azathioprine in paediatric BD patients, although it has been widely and successfully used for uveitis and vascular and neurological involvement [55,58].

Regarding alternative treatment options, anakinra (IL-1 receptor antagonist) [70] and ustekinumab (anti-IL-12/23) [71] were used in trials in adult BD patients; orogenital ulcers showed a partial response to anakinra, and ustekinumab use resulted in the amelioration of both mucocutaneous lesions and joint symptoms [71].

### 6.2. Management of Ocular Involvement

Ocular involvement in BD may be sight threatening, and uncontrolled or underdiagnosed uveitis may result in permanent loss of visual acuity and blindness; hence, it should be recognised and treated promptly in collaboration with an experienced ophthalmologist to achieve early remission [64]. EULAR recommendations suggest that any patient with BD and inflammatory posterior uveitis should be treated with either azathioprine, ciclosporin, interferon-alpha or monoclonal anti-TNF-alpha antibodies, and systemic glucocorticosteroids should not be used alone to control eye involvement [64]. Although EULAR recommendations are based on adult experience, since the evidence is limited to case series in children, they can be extrapolated to paediatric BD patients. Randomised controlled trials demonstrated that both azathioprine and ciclosporin are effective in preserving visual acuity and preventing relapses [69,72]. The successful use of interferon-alpha [73] and anti-TNF agents such as infliximab [74,75] and adalimumab [76] has been reported in refractory cases. In terms of anti-TNF choice, a relatively recent comparative study in 177 BD patients with refractory uveitis showed that both infliximab and adalimumab led to favourable outcomes during a one-year follow-up; however, improvement in ophthalmic examinations was more remarkable after one year of treatment with adalimumab [77]. Case reports and retrospective case series in children all reported favourable outcomes in BD uveitis with anti-TNF use, especially with adalimumab [78,79]. In a retrospective case report of 34 paediatric BD patients, azathioprine in addition to glucocorticosteroids was the treatment of choice for 10/12 children with uveitis [55].

An RCT with gevokizumab (IL-1-regulating monoclonal antibody) [80] and secukinumab (anti-IL-17) [81] did not meet their primary end points. Tocilizumab demonstrated promising outcomes in five adult BD cases with refractory uveitis who previously failed to respond to IFN-alpha and anti-TNF treatment [82].

Treatment choices should be individualised depending on patient, physician and health-system-related factors, such as patient age and sex, organ involvement, susceptibility to infections, tolerability, physician experience and funding for specific medications [14,64], since there are no trials that demonstrate the superiority of any of the aforementioned treatments.

### 6.3. Management of Other Major Organ Involvement and Vascular Disease

Other major organ involvement in BD includes neurological, gastrointestinal and vascular manifestations.

From a general perspective, EULAR recommendations suggest starting treatment with high-dose corticosteroids with a slow weaning plan, in addition to the use of non-biologic immunosuppressive agents such as azathioprine or ciclosporin for major organ involvement, and escalation to TNF inhibitors for more severe and refractory cases [64]. There are some exceptions and additional important aspects that need to be considered for different organ involvement. For instance, ciclosporin should be avoided in neurological involvement since it was shown to increase the risk of CNS involvement in different adult case series, possibly via an off-target endothelial toxicity effect [83]. Limited observational studies with a small number of patients demonstrated the successful use of IL-6 blockade (tocilizumab) for the neurological involvement of BD. Parenchymal involvement is different from cerebral venous thrombosis, as the latter is assessed as a vascular manifestation, and an acute CVST can be treated with a high dose of corticosteroids and a short course of anticoagulation [84]. In a Turkish retrospective case series of 12 paediatric BD patients with CVST [58], all patients received intravenous pulse methylprednisolone followed by slow tapering of oral prednisolone; 11/12 were concomitantly started on azathioprine, and 12/12 received anticoagulation (11/12 with enoxaparin sodium and 1/12 with warfarin sodium). Only one patient who did not receive azathioprine relapsed at the second year of follow-up [58].

The management of vascular manifestations involves immunosuppressive agents and anticoagulation, although controversy remains for the latter, as thrombosis is a consequence of vessel wall inflammation rather than a hypercoagulable state [64]. Treatment modalities for acute deep vein thromboses include glucocorticosteroids alongside immunosuppressives such as azathioprine, ciclosporin or cyclophosphamide [64]. For refractory cases, TNF blockade with the consideration of adding an anticoagulant agent was recommended, provided that the bleeding risk is low and pulmonary artery aneurysms are ruled out [64]. The precise role of immunosuppressive options and anticoagulation is still largely debated, though study outcomes favour the use of immunosuppressants over anticoagulation for venous thromboses of BD [85]. In the largest cohort of adult BD cases with 296 venous thromboses, multivariate analysis showed that the use of immunosuppressive treatment and glucocorticosteroids prevented relapses [85]. Further, 3/4 (75%) patients in the anticoagulant-treated group developed new thromboses whilst on treatment, compared to 2/16 (12.5%) in the immunosuppressant-treated group. Ahn et al. [86] compared the use of immunosuppressive therapy alone versus immunosuppressive therapy and anticoagulation, and no significant difference between thrombosis recurrence rates was found between the two groups.

Although extremely rare in paediatric BD, high dose glucocorticosteroids and cyclophosphamide are recommended for the management of pulmonary artery or cardiac aneurysms in the updated EULAR consensus, with escalation to TNF inhibitors for refractory cases [64].

For the gastrointestinal manifestations of BD, EULAR recommendations suggest the exclusion of other possible gastrointestinal pathologies (including ulcers), infections (including tuberculosis) and inflammatory bowel disease, since gastrointestinal symptoms can generally be non-specific and abdominal pain is usually the most common presenting symptom in children [9,64]. Acute exacerbations of gastrointestinal symptoms in BD can be managed with glucocorticosteroids and disease-modifying anti-rheumatic agents [87]. The use of 5-ASA and sulphasalazine has been reported to be successful in milder presentations; however, azathioprine should be the next treatment option for more severe cases, and in azathioprine non-responsive or refractory cases, escalation to an anti-TNF-alpha agent and/or thalidomide should be considered [87,88]. Gastrointestinal complications such as major bleeding, perforation and obstruction may occur; hence, timely recognition and surgical consultation are crucial in these cases [64].

## 7. Mimics of BD

### 7.1. Monogenic Mimics of Paediatric BD

#### 7.1.1. Haploinsufficiency of A20 (HA20)

Zhou et al. [13] reported on five families of different ethnicities with autosomal dominant, high-penetrance mutations in *TNFAIP3* (Table 2), causing a hereditary systemic autoinflammatory disease via impairment of the regulatory function of the A20 protein. HA20 patients presented with Behçet-like phenotypes, including oral and genital ulcers, pathergy, vascular thrombosis and neurological and gastrointestinal involvement (Table 3). Indeed, whole-exome sequencing in familial BD patients was able to identify loss-of-function mutations in *TNFAIP3* [89]. The wild-type A20 protein was shown to suppress the NF-κB pathway, as opposed to the mutated A20 protein, which resulted in higher NF-κB signalling activity and thus increased transcription of genes encoding proinflammatory cytokines such as IL-1β, IL-6 and TNF-alpha. A20 also functions as a negative regulator of the NLRP3 inflammasome, independent of its role in the NF-κB pathway [13]. This might explain the activation of caspase-1, the increased production of active IL-1β and IL-18 and hence the autoinflammatory phenotype of the condition. HA20 should be considered in patients with a family history of BD, early onset of symptoms and recurrent febrile disease [33,90]. Thus far, HA20 patients with various organ and system involvement have been reported. For instance, a patient treated for longstanding intestinal BD-like symptoms was later diagnosed with A20 haploinsufficiency [91].

#### 7.1.2. Otulipenia (Loss-of-Function Mutations in OTULIN)

Zhou et al. [92] reported a BD-like autoinflammatory disease phenotype in four affected patients with neonatal-onset fever, neutrophilic dermatosis/panniculitis and failure to thrive but without a primary immunodeficiency. Loss-of-function mutations were detected in *OTULIN (FAM105B),* a protein playing a role in the regulation of innate immune signalling complexes, mainly NF-KB. Affected patient fibroblasts and peripheral blood mononuclear cells (PBMCs) carrying mutated *OTULIN* showed a substantial defect in the deubiquitination of the target molecules, causing enhanced activation of the *NF-KB* signalling pathway and thus an increase in proinflammatory cytokines that can delineate the autoinflammatory phenotype [92]. The identification of loss-of-function mutations in *OTULIN* has provided further insight into the emerging spectrum of ubiquitin pathway-associated autoinflammatory conditions, along with A20 haploinsufficiency [92,93].

#### 7.1.3. Deficiency of Adenosine Deaminase-2 (DADA-2)

In 2014, DADA-2 was described as a novel monogenic autoinflammatory condition with early-onset vasculopathy, lacunar strokes, livedoid rash and other neurovascular manifestations [94]. Following this, the identification of patients and further insight into the condition revealed a presentation with haematological involvement and immunodeficiency [95]. Subsequently, case series carrying *ADA2* mutations and displaying a novel combination of Behçet-like manifestations were reported [96] (Table 3). In the same report involving three families, two sisters from the same family were initially diagnosed with BD and had a partial response to treatment; an *ADA2* mutation was subsequently identified using next-generation sequencing [96]. One sibling presented with genital ulcers, recurrent fever and arthralgia and developed vertigo and erythema nodosum during the disease course, while the other sibling presented with recurrent oral aphthae and erythema nodosum [96].

#### 7.1.4. Other Systemic Autoinflammatory Syndromes (SAIDs) and Periodic Fever Syndromes

Several typical features of BD overlap with those of SAIDs; therefore, these syndromes should be considered in differential diagnoses [97] (Table 3). For instance, cryopyrin-associated periodic syndrome (CAPS) and mevalonate kinase deficiency/hyperimmunoglobulin D syndrome may both present early in life with oral ulceration and gastrointestinal symptoms [97]. Similarly, the presence of the familial Mediterranean fever gene mutation (*MEFV*) Met694Val was found to be associated with an increased frequency of BD in the Turkish population [29]. Uveitis represents another example of overlapping features, as it can also be seen in CAPS, TRAPS and Blau syndrome.

#### 7.1.5. Primary Immunodeficiencies (PID)

1. Chronic granulomatous disease (CGD)

CGD is a monogenic PID characterised by an impaired oxidative burst reaction that leads to inadequate neutrophil phagocytosis in response to bacterial and fungal infections [98]. Typical features include abscesses and granulomatous lesions of the skin, lungs, lymph nodes and liver [99]. Patients can present with mucocutaneous lesions, which might lead to the consideration of other differential diagnoses. In a recent case report of a 9-year-old-girl with recurrent oral aphthae and non-specific gastrointestinal symptoms, unusual infectious complications during the disease course led to genetic analysis revealing a characteristic mutation for CGD [98]. Papadopoulou et al. also described a case with a compound heterozygous mutation in *NCF-1* that caused CGD in their monogenic BD mimic cohort [33].

2. PID caused by mutations in the NF-KB signalling pathway

**Figure 2:**
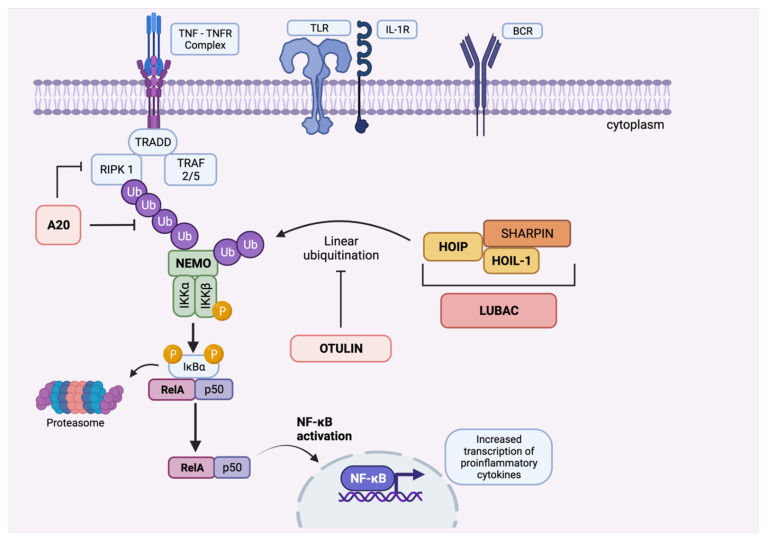
Overview of the canonical NF-κB signalling pathway and the role of A20 and OTULIN proteins (created by Biorender). Mutations in proteins that can result in BD-like phenotypes are shown in bold. Induction of the pathway by Toll-like receptors (TLRs), T-cell receptors (TCRs), B-cell receptors (BCRs) and cytokine receptors (TNFα, IL-1β, IL-6, etc.) initiates a cascade, followed by the stimulation of IκB kinase (IKK) complex, which consists of three subunits: IKKα, IKKβ, and IKKγ (NEMO, NF-κB essential modulator). Activation of IκBα via phosphorylation leads to release of NF-κB1, which is essentially a dimer composed of RelA/p65 and p50. RelA/p50 complex subsequently translocates to the nucleus, leading to activation of the NF-κB pathway, regulation of genes and, ultimately, transcription of proinflammatory cytokines. Linear ubiquitin chain assembly complex (LUBAC) that consists of HOIL-1, HOIP and SHARPIN activates NF-κB pathway by ligation of linear ubiquitin chains to NEMO and RIP1. OTU deubiquitinase with linear linkage specificity (OTULIN) functions as a deubiquitinating enzyme that catalyzes linear ubiquitin chains from proteins modified by the LUBAC, thereby controlling overactivation of the NF-κB pathway. Similarly, A20 protein is also shown to contain deubiquitinase and E3 ligase domains, which also results in deubiquitination of NF-κB upstream proteins, hence negatively regulating inflammation. These findings may elucidate the mechanisms by which mutations in A20 and OTULIN result in overactivation of the NF-κB signalling pathway and, consequently, enhanced inflammatory response. Abbreviations: IKKα and IKKβ, inhibitor of nuclear factor kappa kinase subunit α and β; LUBAC, linear ubiquitin chain assembly complex; NEMO, NF-κB essential modulator; NF-κB, nuclear factor kappa-B; RIPK1, the death domain-containing protein kinase receptor-interacting protein 1; TNFα, tumour necrosis factor-alpha; TNFR, tumour necrosis factor receptor; TRADD, tumour necrosis factor receptor-1-associated death domain protein. TRAF 2/5:TNF receptor-associated factor 2 and 5.

Several monogenic diseases associated with PIDs have recently been discovered and reported to display BD-like phenotypes [100]. Most of these associated genes, identified via whole-exome (WES) and whole-genome sequencing studies, were found to have a role in the NF-KB signalling pathway, which might shed light on associations with BD as one of the aetiopathogenetic mechanisms [100] (Figure 2). Mutations in *TNFAIP3* that impair the normal functioning of this pathway were previously discussed. In the following section, we focus on other mutations that play a role in NF-KB dysregulation, some of which are known to cause NF-KB-related autoinflammatory diseases displaying BD-like features [97].

i. NEMO (NF-κB essential modulator)

X-linked mutations in *NEMO* are known to be associated with incontinentia pigmenti (IP) in females and hypomorphic NEMO deficiency syndrome in males. [101]. Heterozygous mutations in *NEMO* were found to be associated with familial BD [102] and the concurrence of BD in two different IP cases [103]. Autoinflammatory features in NEMO deficiency, such as arthritis, colitis and dermatitis, were attributed to the failure of NEMO to recruit the A20 protein, thereby resulting in the loss of inhibition of the pathway [104].

ii. NF-κB1 (nuclear factor kappa B)

Polymorphisms of *NFKB1* were thought to be associated with BD, particularly in ocular involvement [105]. Yenmis et al. supported this hypothesis by demonstrating an increased risk of BD in Turkish patients with polymorphisms in this gene [106]. Following this, Kaustio et al. reported three unrelated Finnish patients who presented with characteristic features of BD and were eventually found to have heterozygous mutations in *NFKB1* that gave rise to distinct immunological phenotypes [107].

iii. RELA (p65) (relative v-rel reticuloendotheliosis viral oncogene homolog A)

Using WES, Adeeb et al. recently identified five familial cases of a novel *RELA*-truncating mutation that resulted in BD-like mucocutaneous ulcerations and neuromyelitis optica in three generations of an Irish family [108]. Data from this case series indicated that loss-of-function mutations in *RELA* impaired NF-κB signalling and increased apoptosis, causing the BD-like phenotype [108] (Figure 1C).

3. Periodic fevers with immunodeficiency and thrombocytopenia (PFIT)

PFIT is a recently described hereditary autoinflammatory syndrome caused by a mutation in the actin regulatory gene, *WDR1* [109]. WDR1 and actin regulation play key roles in the activation of the inflammasome pathway in autoinflammation [109]. Standing et al. described two Pakistani girls born to consanguineous parents presenting with severe autoinflammation, including early-onset fevers, recurrent perianal ulceration, recurrent oral aphthae causing scarring and microstomia in one of them [109]. Familial BD was the initial diagnosis for both; however, a homozygous missense mutation in actin regulatory *WDR1* identified via WES led to a confirmed diagnosis of PFIT [33,109].

#### 7.1.6. Trisomy 8

BD has increasingly been recognised in association with constitutional trisomy 8 mosaicism and acquired trisomy 8 secondary to myelodysplastic syndromes in adults [110,111]. Activation of the NF-κB pathway might be the underlying pathology for the BD-like phenotype in these patients [112].

#### 7.1.7. Fabry Disease (Lysosomal Storage Disease)

Fabry disease is an X-linked lysosomal storage disorder associated with mutations in the *GLA* gene that result in the deficiency of a lysosomal enzyme, alpha-galactosidase A, thereby causing abnormal accumulation of glycosphingolipids in lysosomes, which is responsible for the clinical phenotype [113]. In their recent report, Papadopoulou et al. described one patient who was initially diagnosed with paediatric BD with fever of unknown origin, recurrent oral ulceration, myalgia, colitis and a skin rash reminiscent of panniculitis [33] (Table 3). Despite treatment with corticosteroids and cyclophosphamide and maintenance with azathioprine, cerebral venous thromboses occurred, which prompted genetic testing, and WES revealed a mutation in *GLA* that confirmed the diagnosis of Fabry disease [33].

#### 7.1.8. Other Monogenic Mimics of BD

In addition to the abovementioned monogenic mimics of BD, Papadopoulou et al. reported one more case with a clearly pathogenic variant in the *AP1S3* gene, which is classically associated with pustular psoriasis and deranged trafficking of Toll-like receptor 3 [33]. Furthermore, in the same case series, two further cases with likely pathogenic variants in other genes were described [33]. Mutation in the tyrosine-protein kinase *LYN* gene was detected in one case who presented with fever, recurrent mouth ulcers, panniculitis, testicular and abdominal pain. In the other case, a novel *STAT1* gain-of-function variant was identified, consistent with the diagnosis of chronic mucocutaneous candidiasis [33].

### 7.2. Non-Monogenic Mimics of BD

The list of differential diagnoses in BD is broad. Viral, fungal and bacterial infections, including tuberculosis, should always be considered, especially with mucocutaneous, gastrointestinal and ocular involvement. Monogenic autoinflammatory syndromes are discussed above as important differentials; additionally, non-monogenic periodic fever syndromes, such as periodic fever, aphthous stomatitis, pharyngitis and cervical adenitis syndrome (PFAPA), can masquerade as BD in infancy and early childhood [114]. Conversely, patients initially diagnosed with PFAPA may ultimately declare themselves as having BD later in life, since the immunopathogenesis somewhat overlaps between these two diseases [115]. Manthiram et al. identified genetic similarities in BD, PFAPA and recurrent aphthous stomatitis—with stronger HLA associations in more severe cases—and proposed naming these conditions ‘Behçet’s spectrum disorders’ [115].

Prothrombotic conditions must also be remembered and investigated in those presenting with thromboses. Other non-monogenic mimics of BD are considered below.

#### 7.2.1. Inflammatory Bowel Disease (IBD)

BD significantly overlaps with IBD, as both conditions present with gastrointestinal symptoms, oromucosal ulcerations, skin manifestations, uveitis and joint involvement [116]. EULAR recommendations suggest excluding possible underlying pathologies, including IBD, NSAID ulcers and tuberculosis, before attributing symptoms to intestinal BD [64]. Gastrointestinal manifestations of BD were shown to be more common in the Far East, especially in Japan, but rather infrequent in Eastern Mediterranean [117]. Symptoms of non-specific abdominal pain and diarrhoea resemble Crohn’s disease, and BD-associated ulcers generally occur in the terminal ileum [14]. Vascular involvement with Budd–Chiari syndrome, bleeding and perforation can complicate intestinal BD [14]. Mucosal biopsies in BD demonstrate chronic or active mucosal inflammation, as well as vasculitic findings, which can help distinguish the condition from IBD [14]. Of note, anorectal disease and granuloma formation are considerably more specific to Crohn’s disease [25]. Genital ulcers are not seen in IBD, although perineal fistula in Crohn’s disease may be mistaken for genital ulceration.

#### 7.2.2. Seronegative Spondyloarthropathies (SpA)

As mentioned, BD was initially included in the spondyloarthropathy group because of joint and gastrointestinal involvement. However, it was then understood that, unlike SpA, there was no association with HLA-B27 in BD patients; instead, the majority of BD patients were carriers of HLA-B51, and axial skeletal arthritis was not a prominent feature [14]. Recently, controversy regarding the classification of BD under SpA has arisen for two main reasons: firstly, there may be a common immunopathological pathway involved in BD, ankylosing spondylitis (AS) and psoriatic arthritis (PsA), such as IL-10, IL-17 and IL-23; secondly, the acne–arthritis–enthesopathy cluster of BD is associated with SpA-related symptoms [14]. As previously discussed, *ERAP-1* gene polymorphisms have been found to be associated with MHC class I and reported as susceptibility loci for AS and PsA. Subsequently, since BD, AS and PsA are associated with variants in *HLA* encoding MHC class I, these conditions are now described by some as ‘MHC-I-opathies’ [14].

#### 7.2.3. Other Vasculitides

Vasculitis in BD may mimic other types of inflammatory vasculitides since it may involve vessels of any size and type [4]. Nevertheless, venous involvement is a unique feature of BD vasculitis, and there is no necrotizing vasculitis in small vessels as in anti-neutrophil cytoplasmic antibody-associated vasculitides, nor are there immune complexes [118]. The pulmonary artery aneurysms seen in BD can also be observed in Takayasu arteritis, although they are of particular prognostic significance in BD since they are associated with poorer outcomes [97].

Hughes–Stovin syndrome (HSS) is another extremely rare systemic vasculitic disease that has also been considered a severe variant of BD. The condition is characterised by widespread deep vein thromboses and pulmonary and/or bronchial artery aneurysms with high morbidity and mortality rates [119]. In the context of disease severity and a few paediatric case reports [120,121], HSS should also be considered in the young.

#### 7.2.4. Neutrophilic Dermatoses

Neutrophilic dermatoses are a heterogeneous group of non-infectious systemic diseases characterised by non-neoplastic neutrophilic inflammatory infiltration of the skin and frequently associated with multisystemic diseases [122]. BD, pyoderma gangrenosum and acute febrile neutrophilic dermatosis (Sweet’s syndrome) can be included in this group, and they may all demonstrate a pathergy reaction [122].

## 8. Comparison of Paediatric and Adult-Onset BD

The expression of symptoms has been shown to differ between children and adult patients in comparison studies: in general, non-specific gastrointestinal, neurological symptoms, arthralgia and a positive family history are more pronounced in the paediatric age group; genital ulceration, ocular symptoms and vascular involvement are more widely described in adults [6,7,123]. Disease activity scores and severity indexes appear to be lower in children as compared to adults, although this might be attributed to a shorter disease course rather than quiescent disease activity [7,8].

## 9. Conclusions

Emerging evidence on monogenic mimics of BD has emphasised the importance of early genetic testing, particularly for those with early-onset, atypical features and familial aggregation, to exclude monogenic diseases masquerading as BD. NGS can also enable HLA typing, thus effectively providing the opportunity to classify BD as typical (i.e., HLA-B51 with no monogenic mutations) or a monogenic BD mimic. Advances in genomics will presumably lead to the identification of further novel variants that might explain BD-like presentations and the marked diversity in symptoms. International collaboration is needed for randomised controlled trials in paediatric BD to scrutinise heterogeneous symptom expression in different geographical regions and to establish nuanced consensus guidelines for diagnosis and management in the paediatric population.

## Figures and Tables

**Table 1:** Comparison of different international criteria for Behçet’s disease.

International Criteria	ISG	ICBD *	PED-BD Criteria **
		Value/Item	Value/Item
Recurrent oral aphthosis	+ Mandatory (at least 3 attacks/year)	+2	+1 (at least 3 attacks/year)
	Plus at least 2 of		
Genital ulceration	+ or aphthosis (at least once)	+2	+1 (typically with scar)
Skin lesions	+	+1	+1
Ocular lesions	+	+2	+1
Positive pathergy test	+	+1	N/A
Neurological signs	-	+1	+1 (with the exception of isolated headaches)
Vascular signs	-	+1	+1 (venous thrombosis, arterial thrombosis, arterial aneurysm)

ISG: International Study Group of BD 1990, ICBD: International Criteria for BD 2014 (* BD diagnosis can be made if total score is ≥ 4), PED-BD: paediatric criteria for BD 2015 (** 3/6 items are required for a diagnosis of paediatric BD) (+/-): presence or absence of criterion. N/A: not applicable.

**Table 2:** Monogenic mimics of BD with affected gene and protein.

Monogenic Mimics of BD			
Disease	Affected Gene	Protein	Role of Protein
Systemic autoinflammatory diseases (SAIDs)			
Haploinsufficiency A20	*TNFAIP3*	A20	Negative regulation of inflammation via NF-κB pathway
DADA-2	*ADA-2*	ADA-2	Regulation of cell proliferation and differentiation
FMF	*MEFV*	Pyrin	Regulation of pyrin–inflammasome complex via caspase-1
Blau syndrome	*NOD2*	NOD2	Regulation of innate immunity signalling via NF-κB pathway
Otulipenia	*OTULIN*	Otulin	Regulation of innate immunity signalling via NF-κB pathway
Primary immunodeficiencies (PIDs)			
CGD	*CYBB, NCF-1, NCF-2, NCF-4*	Components of NADPH oxidase	Production of reactive oxygen species in phagocytes
NEMO	*NEMO*	NF-κB essential modulator	NF-κB signal modulation
NFKB1	*NFKB1*		Regulation of innate immunity signalling via NF-κB pathway
RELA (p65)	*RELA*	RELA	Regulation of innate immunity signalling via NF-κB pathway
PFIT	*WDR1*	WD40 repeat protein	Activation of inflammasome pathway via actin regulation
Others			
Trisomy 8	N/A	N/A	Effect on NF-κB pathway
Fabry Disease	*GLA*	Alpha-galactosidase A	Degradation of glycosphingolipids in lysosomes

*ADA-2*, adenosine deaminase-2; BD, Behcet’s disease; CGD, chronic granulomatous disease; *CYBB*, cytochrome B-245 beta chain; DADA-2, deficiency of adenosine deaminase-2; FMF, familial Mediterranean fever; *GLA*, α-galactosidase A gene; *MEFV*, Mediterranean fever; NADPH, nicotinamide adenine dinucleotide phosphate; N/A: not applicable; *NCF*, neutrophil cytosolic factor; *NEMO*, NF-κB essential modulator; *NF-κB*, nuclear factor kappa-B; *NOD2*, nucleotide-binding oligomerization domain containing 2; *OTULIN*, OTU deubiquitinase with linear linkage specificity; PFIT, periodic fevers with immunodeficiency and thrombocytopenia; *RELA*, relative v-rel reticuloendotheliosis viral oncogene homolog A; *TNFAIP3*, tumour necrosis factor-alpha-induced protein 3; *WDR1*, WD repeat-containing protein 1.

**Table 3:** Comparison of clinical manifestations in BD and monogenic mimics.

Organ/System Involvement	BD	HA 20	Blau Syndrome	DADA-2	FMF	Otulipenia	CGD	NEMO	NF-κB	RELA	PFIT	Trisomy 8	Fabry Disease
Recurrent oral aphthosis	√	√	-	-	-	-	√	√	√	√	√	√	-
Genital ulceration	√	√	-	-	-	-	√	*	√	√	√	√	-
Ocular	√	√	√	-	-	*	-	√	√	-	-	*	√
Skin lesions	√	√	√	√	√	√	√	√	√	√	√	√	√
Pyrexia	√	√	√	√	√	√	√	√	√	√	√	*	*
Vascular	√	√	*	√	-	√	-	-	√	-	-	√	√
Neurological	√	√	*	√	-	√	-	-	√	√	*	*	√
Gastrointestinal	√	√	*	√	√	*	√	√	√	*	√	√	√
Arthralgia/arthritis	√	√	√	√	√	√	-	√	√	*	*	√	√
Systemic inflammation	√	√	√	√	√	√	-	√	√	√	√	-	-
Immunodeficiency	-	*	*	√	-	-	√	√	√	√	√	-	-
Haematological	-	√	-	√	-	-	*	√	-	-	√	√	√

Different clinical features observed in BD and monogenic mimics. BD, Behcet’s disease; CGD, chronic granulomatous disease; DADA-2, deficiency of adenosine deaminase-2; FMF, familial Mediterranean fever, HA 20, A20 haploinsufficiency; NEMO, NF-κB essential modulator; NF-κB, nuclear factor kappa-B; PFIT, periodic fevers with immunodeficiency and thrombocytopenia; RELA, relative v-rel reticuloendotheliosis viral oncogene homolog A. (*) Clinical feature has not been reported in the literature but may be associated. (-) Clinical feature has not been reported in the literature and is unlikely to be observed for that diagnosis.

## Data Availability

No new data were created or analyzed in this study. Data sharing is not applicable to this article.
